# Predictors of Excessive Daytime Sleepiness in Medical Students: A Meta-Regression

**DOI:** 10.3390/clockssleep1020018

**Published:** 2019-04-11

**Authors:** Haitham Jahrami, Hajar Alshomili, Noora Almannai, Noora Althani, Adel Aloffi, Haifa Algahtani, Cary A. Brown

**Affiliations:** 1 Ministry of Health, Juffair 340, Kingdom of Bahrain; 2 College of Medicine and Medical Sciences, Arabian Gulf University, Manama 329, Kingdom of Bahrain; 3 Department of Occupational Therapy, Faculty of Rehabilitation Medicine, University of Alberta, Edmonton, AB T6G 2G4, Canada

**Keywords:** sleepiness, university students, Epworth sleepiness scale, medical school

## Abstract

Excessive daytime sleepiness (EDS) is highly prevalent among medical students and can have serious negative outcomes for both students and their patients. Little is known about the magnitude and predictors of EDS among medical college students. A meta-regression analysis was conducted to achieve these two targets. A systematic search was performed for English-language studies that reported the prevalence of EDS among medical students using the Epworth sleepiness scale (ESS), age, sex, sleep duration and sleep quality as predictive variables. A total of nine observational studies (*K* = 9, *N* = 2587) were included in the analyses. Meta-regression analyses were performed using mean age (years), sex (proportion of male subjects), sleep duration (hours/night) and sleep quality index score (continuous scale) as moderators for EDS—with the prevalence of EDS as an outcome variable. An interaction term of sleep duration X sleep quality was created to assess if these two variables simultaneously influenced the outcome variable. Utilizing the ESS, the pooled prevalence of EDS among medical students was 34.6% (95% Confidence Interval (CI) 18.3–50.9%). Meta-regression models of age, sex, sleep duration and sleep quality alone revealed poor predictive capabilities. Meta-regression models of sleep duration–sleep quality interaction revealed results with high statistical significance. The findings from this review contribute supporting evidence for the relationship between sleep duration and sleep quality scores (i.e., sleep duration X sleep quality score) in predicting EDS in medical students.

## 1. Introduction

Excessive daytime sleepiness (EDS) is a significant health problem [1] with serious consequences, including accidents and injury to self and others [2,3], reduced occupational performance at work or school [4,5], impaired cognitive and social functioning [6], poor physical health [7,8] and increased risk of dementia later in life [9]. For caring professionals, such as physicians and medical students, the negative impact of EDS effects not only the individual but also the safety and quality of care of patients [10,11,12]. 

The working definition of EDS in this review is adapted from Mahmood and colleagues [13], and is a state ranging from mild daytime drowsiness to falling asleep excessively throughout the daytime—characterized by the inability to stay awake, alert and optimally functional throughout the day [13]. 

EDS emerges from the combination of one or more behavioral factors [14] (e.g., altered sleep phase, insufficient sleep), environmental factors [1] (e.g., room temperature), medical factors [15] (e.g., otolaryngologic and respiratory problems, neurological problems, psychiatric problems and other organic diseases), dietary and medications factors [16] (e.g., smoking, alcohol or psychotropic medications). Frequently, however, there may not be a discrete, identifiable cause and the only diagnosis possible is that of idiopathic EDS. 

Estimating the prevalence of self-reported EDS has proven to be challenging [1]. This is partly due to the various definitions offered for EDS by the developers of different measurement tools. There is a small group of standardized and psychometrically sound tools that have been used most commonly in EDS research, including the Epworth sleepiness scale (ESS), Stanford sleepiness scale, Karolinska sleepiness scale, Swiss narcolepsy scale [17] and, more recently, the Flinders fatigue scale [18]. 

Whilst there is an inconsistency in researchers’ selection of EDS measurement tools, there is a clear agreement that the magnitude of EDS is high. Indeed the prevalence of EDS has been reported to range from 10% [19] up to 36% in the adult general population [20]. It is important to note that university students are a cohort who appear to be even more susceptible to EDS, with approximately 50% of university students in some studies reporting they are overly tired, drowsy or sleepy [20,21]. As noted previously, EDS has serious negative consequences for both healthcare students and their patients, and research finds that up to 63% of medical students experience EDS—which is markedly concerning [21]. 

Obtaining an accurate, reliable estimate for the magnitude of the problem is crucial to both identifying the risk factors associated with the problem and to developing EDS prevention and treatment strategies specific to the context and needs of this population. Accordingly, the following systematic review and meta-analysis were conducted to: (1) obtain a pooled estimate of EDS among medical students and (2) examine potential variables as predictors of EDS. Specifically, the aim of this meta-regression analysis is to study the association between the prevalence of EDS in medical students and their age, sex, sleep duration and sleep quality to expand our knowledge about the impact of these possible risk factors. Based on the previous research literature we hypothesized the following: (1) the pooled estimate for EDS among medical students will be greater than one out of three students and (2) the risk for EDS will be directly associated with sleep duration and/or sleep quality. 

## 2. Materials and Methods

This review and associated analyses were conducted congruent with the guidelines presented in the preferred reporting items for systematic reviews and meta-analyses (PRISMA) statement [22]. 

### 2.1. Search Strategy

The review team formulated a search strategy that included the following keywords: ‘medical students’ AND ‘excessive daytime sleepiness’ OR ‘sleepiness’ OR ‘sleep disturbance’ OR ‘sleep problems’ OR ‘sleep quality’ OR ‘sleep duration’ OR ‘sleep disorder’ OR ‘sleep habit’ OR ‘sleep hygiene’. The search included papers published from the launch of the databases until the second week of September 2018. As a cross-check, two members of the team independently conducted the searches using four databases: MEDLINE, EMBASE, ScienceDirect, ProQuest Medical and the search engine Google Scholar. The review team further manually inspected the citations of the identified papers for potential inclusion in the review. 

### 2.2. Inclusion Criteria

The review focused on observational studies that aimed to identify the prevalence of EDS among undergraduate medical students. The purpose of this meta-regression review was to examine the strength of association between the presence or absence of EDS in medical students and the suggested risk factors of age, sex, sleep quality score and sleep duration. Thus, the following inclusion criteria were applied: (1) published as an original, peer-reviewed, English language article; (2) published before the second week of September 2018; (3) focused on undergraduate medical students as a target population; (4) reported sleep quality score measured using the Pittsburgh sleep quality index (PSQI) and/or sleep durations measured using hours per night; and (5) reported the prevalence of EDS measured using the ESS. For comparison, we required all studies to have used the same indicator of EDS. We selected the ESS because our background literature search revealed it to be the most commonly employed measure in both clinical and research settings to date [13]. The Epworth sleepiness scale collects self-report data about the likelihood of falling asleep during eight common, daily situations, such as sitting quietly after lunch, reading or waiting in traffic [14]. The ESS scores range from 0 (no daytime sleepiness) to 24 (the highest level of daytime sleepiness). The ESS developers identify that the cut-off for EDS risk is 10; scores between 10 and 15 indicated mild/moderate EDS and scores 16 and over indicated severe EDS. 

**Figure 1:**
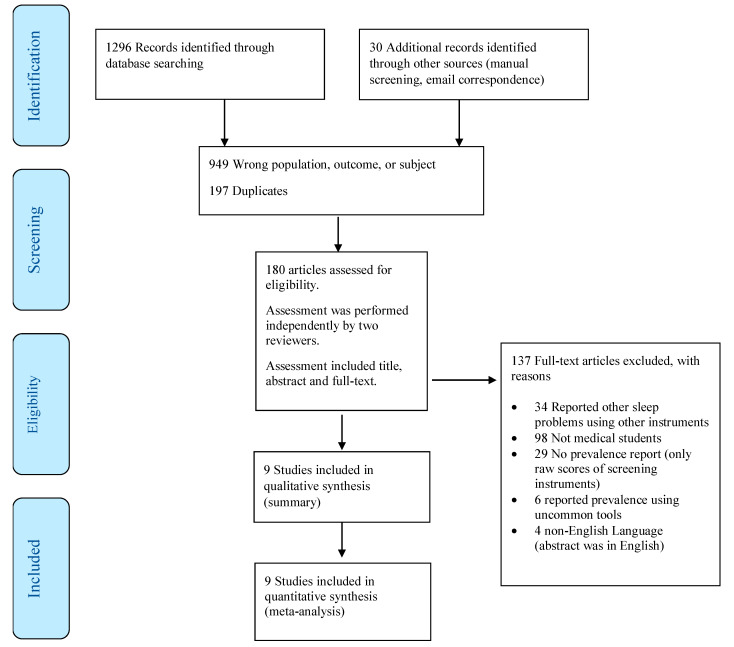
Flow diagram of study inclusion.

We specifically excluded: (1) case reports and case studies and (2) studies that reported the results of medical students combined with non-medical students in the same group, without providing a subgroup analysis. The flow diagram of study inclusion is shown in Figure 1. 

Two members of the review team independently screened titles, abstracts and full-texts, in addition to assessing studies for eligibility criteria and performing the data extraction and research summary. Any disagreements were resolved through discussion with a third reviewer to reach consensus. 

### 2.3. Outcomes and Specific Measures

The anticipated outcome of this systematic review and meta-analysis was to identify predictors of EDS in medical students. The specific aims were to: (1) determine the prevalence of EDS as measured by the ESS among undergraduate medical students and (2) investigate specific variables (age, sex, country, sleep quality index and sleep duration) as predictors of EDS among medical students. 

### 2.4. Data Extraction and Coding

Data were independently extracted by two reviewers and were checked by a third reviewer. The following information was extracted and tabulated systematically: study authors, study country, study period, age, the proportion of male participants, sample size, sleep duration, sleep quality index scores and the main effect of EDS according to ESS. The risk of EDS was measured using continuous scores on ESS as the basis for a dichotomous outcome variable. EDS was estimated in this meta-regression by using the number of cases that screened positive, with an ESS score of ≥10, adjusted for the total sample size. The ESS with a cut-off point of 10 for a mild/moderate risk of EDS has a sensitivity of 93.5% and a specificity of 100% [23]. 

### 2.5. Data Synthesis and Analysis 

The data were pooled in this meta-analysis using a random-effects model using the DerSimonian–Laird method [24], reporting the pooled prevalence and corresponding 95% confidence interval. Meta-regression analysis was performed using mean age (years), sex (proportion of male subjects), sleep quality score (scale data) and sleep duration (hours per night) as independent or predictor variables; and the prevalence of excessive daytime sleepiness as dependent or outcome variable. 

To further examine whether two or more independent variables simultaneously predicted the outcome variable, a statistical interaction variable was created and tested. The interaction variable was defined as sleep quality score × sleep duration. Descriptive statistical analyses were performed using STATA 15.0 [25]. Meta-regressions were performed using OpenMetaAnalyst software provided by the Centre for Evidence Synthesis in Health/Center for Evidence-Based Medicine, School of Public Health, Brown University [26]. 

### 2.6. Ethical Considerations

This review is based on published studies that are available in the public domain; therefore, no ethical approval was necessary to conduct the review. 

## 3. Results

### 3.1. Study Characteristics

Nine studies [27,28,29,30,31,32,33,34,35], involving a total of 2587 respondents from six countries, met the inclusion criteria for the current investigation (see Table 1). The median number of respondents per study was 305 (range 50–576). The median age of the respondents was 21 years (range 20–24 years), the mean sleep quality score was 6.5 and the mean sleep duration was 6.3 hours/night. Approximately 46% of the respondents self-identified as male. 

### 3.2. Prevalence of EDS in Medical Students

Among these medical students, the pooled prevalence of EDS was 34.6% (95% Confidence Interval (CI) 18.3%–50.9%), I^2^ = 99, τ^2^ = 0.06, Q = 0.001. The details of the systematic summary of the studies are presented in Table 1. The raw prevalence estimates of EDS among medical students reported by individual studies ranged from approximately 11% to 63%. Sensitivity analysis demonstrated that individual study affected the overall pooled point prevalence estimate by <1%, suggesting that the overall prevalence estimate is not influenced by any one single study. 

### 3.3. Predictors of EDS in Medical Students

A series of six meta-regression analyses was performed. Results of the analyses revealed that individual factors of age, sex, sleep duration or sleep quality index scores did not appear to account for the risk of EDS among medical students (see Models 1–4 in Table 2). 

Attempting a multiple/complex model including both sleep duration and sleep quality scores also failed statistically to predict EDS among medical students (see Model 5 in Table 2). 

A statistically significant model was obtained when an interaction variable (sleep duration × sleep quality index scores) was added to predict EDS in medical students, with an omnibus *p*-value of 0.001 (see Model 6 in Table 2). 

## 4. Discussion

This review found the prevalence of EDS, among medical students in six countries for which data were derived, was similar to prevalence rates reported by the general population in the same or similar countries. The obtained prevalence rate demonstrates that approximately one out of three medical students self-reported EDS on the ESS to a degree that interferes with their daily functioning. A major outcome of this meta-analysis was that singular differences in age or sex or sleep duration or sleep quality scores alone did not appear to account for the occurrence of EDS in medical students. However, more sophisticated statistical modeling showed that the interaction between sleep duration and sleep quality scores was a highly significant predictor of EDS. 

A statistically significant difference was found between the EDS scores of students categorized as ‘excellent’ and ‘average’ (*p* < 0.001), subjective feelings of insufficient sleep (*p* < 0.001) and sleeping less than 6 hours a night for six consecutive nights (chronic sleep deprivation) (*p* < 0.005) [36]. 

The association among the risk of EDS, sleep duration and sleep quality has been previously raised by a number of studies. Thus, the failure of sleep duration and sleep quality to emerge as predictors for EDS is an unexpected finding for the current work. Based on previous literature, we hypothesized that short sleep duration or poor sleep quality were risk factors that would specifically place students at risk for developing EDS. However, the results suggest that the association between EDS and sleep duration or sleep quality is more complex and is facilitated by other factors. Importantly, the relationship between sleep duration and sleep quality scores suggests there is a developmental path to EDS among medical students. This interaction between sleep duration and sleep quality further strengthens the notion that sleep deprivation is cumulative in nature. For example, sleep deprivation for a period of two weeks may cause significant issues, but is not responsible for EDS. On the other hand, chronic sleep deprivation that interactively affects (or is affected by) poor sleep quality does indeed cause significant daytime sleepiness. This potential bidirectional relationship greatly expands the range of intervention strategies that can be called upon by both individual students and policymakers. Thus, prevention and treatment efforts should address the underlying causes of EDS and promote both sleep duration (quantity) and sleep quality by ensuring good sleep hygiene and sleep-promoting organizational policies (such as housing, noise reduction, hours of study [37] and limiting on-campus access to sleep-inhibiting foods and beverages). Our results suggest that adjustment of sleep duration is important in preventing EDS [38]. For example, utilizing the model coefficients and intercept in estimating the risk of EDS, if we replace the 6.3 hours/night sleep duration we found to be the average across studies included in our review with 7 hours/night (the minimum recommended sleep duration for this age group), the predicted prevalence rate estimate drops significantly to approximately 5%. 

These findings have direct application and suggest that, given the widespread magnitude of the problem, investment in sleep duration education is critically important. Possible intervention areas are to recognize and educate both students and faculty about the sleep-negative impact of non-stop availability of communication systems such as e-mails and instant messaging; the physiological sleep suppression consequences of night-time blue-spectrum light exposure from electronic devices and institutional lighting [39]; how online medical learning resources are available 24/7, coupled with all-night electronic gaming and entertainment programs and apps; and how 24-hour shopping and business websites are encouraging sleep deprivation in university students. 

University students experience disturbances in their circadian cycle because of the stress of the demanding academic environment, which is further compounded by sleep-negative habits such as browsing the internet, watching television and live-streaming shows and the use of alcohol, tobacco and recreational drugs—all habits that are common in this population [5,40]. Medical students may not consider sleep as a top priority in the context of their academic requirements as they tend to reduce their sleeping time to have extra hours for their study and workload. 

Focusing on awareness and interventions to promote sleep duration is the ideal from a practical viewpoint. However, knowing about unhealthy practices is not necessarily sufficient motivation for change [41]. Intervention strategies should be aligned with social marketing principles to ensure they offer compelling and relevant content that lead medical students to a positive conclusion when they weigh the pros and cons of changing behavior. A critically overlooked area is the potential for institutional policy to promote better sleep. For example, student housing that is designed with sound and light controls to prevent physiological sleep suppression [42], closing access to online learning materials at certain hours over the night and not setting assignments submission times at midnight can all serve to passively reduce risk without students needing to actively make difficult choices to prioritize sleep over other competing demands. Additionally, university medical programs should develop or implement existing prevention and treatment programs that contain measures of specific and modifiable risk factors to facilitate problem identification, intervention success and further remedial action. 

The findings of this meta-regression clearly highlight that different risk factors interact with each other to produce an effect. Academic staff in medical schools should be aware of factors that put their students at high risk of daytime sleepiness as it hinders student learning and health, academic and clinical performance and ethical decision-making [43], carrying potentially dangerous consequences for patients and co-workers with medico-legal implications [44,45]. 

This review has several limitations that need to be considered when interpreting the results. First, this review focused on medical students as a target population, and although the findings are expected to be generalizable to other university students, this conclusion is premature in the absence of other reviews. Furthermore, the studies included in this meta-analysis came from only six countries, so results cannot yet be generalized at a global level. Second, the risk factors in this review were few due to the limited availability of common information in the original articles. For example, until the last 10–15 years, in many countries the majority of students were male. However, some studies reveal that EDS varies by gender, usually with women experiencing higher rates [46,47]. The gender divide for medical students has decreased in the last decade, and it is possible that gender-specific risk factors for EDS that were previously masked by the higher proportion of male students will emerge as this trend continues [48]. Future studies that include an examination of gender-related risk factors are warranted in order to build targeted and relevant intervention strategies reflective of the different gendered socio-economic and biological realities of university students [49]. 

Finally, studies including other measures (e.g., the Stanford sleepiness scale) of EDS may reveal different results. However, the ESS continues to be the most widely used measure and excluding other tools was necessary for computational purposes. Future studies are encouraged to include additional relevant variables, e.g., stimulants intake, living environment, electronic device use, dietary behaviors and physical activity. Broadening our understanding of how multiple bio-psycho-social aspects interact to influence restorative sleep will increase opportunities to affect change based on prevention and intervention strategies that medical students find relevant and acceptable. It must also be acknowledged that the interaction analysis gives a purely statistical result and caution should be used in its clinical application. 

This review has some delimitations that cannot be controlled. A major delimitation of this meta-regression is the number of studies with the required information. The current meta-analysis only had nine studies for inclusion, and the outcome of interest is binary in nature, which may require a larger sample size for more robust conclusions. Another delimitation is related to the use of the ESS. Although the ESS is a common and important instrument, it must be acknowledged that the ratings to calculate an EDS score are subjective and rely largely on the subject’s memory, which may be biased. Thus, the ESS could have suboptimal accuracy in a non-clinical sample. Future researchers are encouraged to use additional tools to further advance our knowledge on the topic. 

## 5. Conclusion

To conclude, the results from our analysis provided evidence for the role of the interaction between sleep duration and sleep quality scores (e.g., sleep duration × sleep quality score) in predicting EDS in medical students. This interaction points to the role that chronic sleep deprivation and poor sleep quality play in an increased risk for persistent EDS. Future prospective studies are needed to investigate the effectiveness of simple, acceptable and cost-effective interventions. 

## Figures and Tables

**Table 1:** Summary of the studies examining the prevalence of excessive daytime sleepiness among medical students.

Study	Country	Design; Sampling; Response Rate	Number of Respondents	Number of Cases with EDS ^†^	Age * (years)	Male %	Sleep Duration (hours/night) *	Sleep Quality Index *
Kang and Chen et al., 2009	China	Cross-sectional; Probability; 81.2%	160	23	20.3 ± 1.9	50.62	6.7 ± 1.3	4.9 ± NR
Giri et al., 2013	India	Cross-sectional; Convenient; NR	50	10	22.4 ± 0.5	40.00	6.3 **	6.5 ± NR
Rique et al., 2013	Brazil	Cross-sectional; Convenient; 86.7%	221	93	22.3 ± 3.8	55.66	6.3 **	6.3 ± 2.6
Pagnin et al., 2014	Brazil	Cross-sectional; Convenient; NR	127	80	21.3 ± 2.3	44.88	6.3 **	6.99 ± 3.0
Surani et al., 2015	Pakistan	Cross-sectional; Convenient; 77.7%	504	52	20 ± 1.4	40.48	6.4 ± 1.5	4.9 ± 2.3
Alsaggaf et al., 2016	Saudi Arabia	Cross-sectional; Convenient; 95%	305	118	22 ± 1.3	41.64	5.5 ± 2.0	6.3 ± NR
Saygin et al., 2016	Turkey	Cross-sectional; Probability; 46.8%	337	54	21.3 ± 2.1	42.09	6.6 ± 1.3	9.03 ± 4.21
Ibrahim et al., 2017	Saudi Arabia	Cross-sectional; Probability; NR	576	403	21 ± 1.46	35.76	6.3 **	7.23 ± 2.97
Priya et al., 2017	India	Cross-sectional; Convenient; NR	307	114	20.5 ± NR	76.54	6.3 **	6.3 ± NR

**^†^** Excessive daytime sleepiness; * Mean ± standard deviation; ** not reported—replaced by the pooled value; NR = not reported.

**Table 2:** Meta-regression models of the prevalence of excessive daytime sleepiness among medical students as an outcome variable with selected predictive factors.

Predictive Factor		β (SE)	95% CI	*p*-Value
1	Age (Years)	0.058 (0.080)	−0.100–0.215	0.606
	Intercept	−0.879 (1.704)	−4.218–2.461	0.472
2	Sex (Proportion of male subjects)	0.056 (0.578)	−1.188–1.076	0.923
	Intercept	0.373 (0.283)	−0.181–0.927	0.188
3	Sleep duration (Hours per night)	−0.216 (0.201)	−0.610–0.178	0.282
	Intercept	1.707 (1.268)	−0.778–4.191	0.178
4	Sleep quality index (PSQI ^†^ Scale)	0.059 (0.051)	−0.042–0.160	0.254
	Intercept	−0.029 (0.335)	−0.685–0.627	0.931
5	Sleep duration	−0.224 (0.140)	−0.587–0.140	0.227
	Sleep quality index	0.061 (0.048)	−0.330–0.154	0.205
	Intercept	1.371 (1.199)	−0.980–3.721	0.253
6	Sleep duration	4.756 (1.479)	1.857–7.655	0.001 *
	Sleep quality index	5.245 (1.535)	2.237–8.253	0.001 *
	Sleep duration × Sleep quality index	−0.793 (0.235)	−1.253–−0.333	0.001 *
	Intercept	−31.182 (9.671)	−50.137–−12.228	0.001 *

^†^ Pittsburgh sleep quality index; * Significant at 0.05, SE = standard error.

## Abbreviations

PSQI	Pittsburg sleep quality index
EDS	Excessive daytime sleepiness
ESS	Epworth sleepiness scale
